# Fisetin promotes skin wound healing and inhibits pathological scar formation through modulation of the PI3K/Akt/TGF-β1 signaling axis

**DOI:** 10.3389/fphar.2026.1793847

**Published:** 2026-03-27

**Authors:** Yi Han, Guixin Sun

**Affiliations:** 1 Department of Traumatic Surgery, Shanghai East Hospital, School of Medicine, Tongji University, Shanghai, China; 2 Department of Traumatic Orthopedics and Joint, People’s Hospital of Guangming District, Shenzhen, Guangdong, China

**Keywords:** collagen remodeling, fisetin, hypertrophic scar, network pharmacology, wound healing

## Abstract

**Background:**

During the healing of skin wounds, excessive fibrosis and collagen remodeling disorders often lead to the formation of proliferative scars. Fisetin is a naturally occurring flavonoid compound with antioxidant and anti-fibrotic properties. However, its therapeutic effect and mechanism of action in wound healing and inhibition of scar formation are still unknown.

**Methods:**

This study used network pharmacological methods to identify potential targets and pathways related to wound healing and proliferative scar formation. Through protein-protein interaction analysis, Gene Ontology analysis and enrichment analysis of the Kyoto Encyclopedia of Genes and Genomes (KEGG), its potential biological functions were clarified. In addition, molecular docking was also carried out to evaluate the binding affinity between fisetin and the core target. The effect of fisetin on wound closure and collagen deposition was evaluated using the full-thickness skin wound model in rats. In in vitro experiments, human dermal fibroblasts were used to study the effect of fisetin on collagen expression and the phosphatidylinositol 3-kinase (PI3K)/Akt signaling pathway.

**Results:**

Network pharmacological analysis highlighted serine/threonine kinase 1 (AKT1) as a central target linking fisetin to wound repair and fibrosis. Functional enrichment indicated significant involvement of the PI3K/Akt pathway. Molecular docking further confirmed a strong binding affinity between fisetin and AKT1. *In vivo* experiments demonstrated that fisetin significantly accelerated wound closure, reduced inflammatory cell infiltration, and improved histological organization. Quantitative analysis showed decreased histological inflammation scores and a reduced the ratio of type I collagen (COL1A)/type III collagen (COL3A), indicating improved collagen remodeling and attenuated scar formation. Moreover, fisetin upregulated PTEN expression while suppressing transforming growth factor-beta1 (TGF-β1) and alpha-smooth muscle actin (α-SMA) expression in wound tissues. *In vitro* experiments further confirmed that fisetin inhibited profibrotic marker expression and regulated PI3K/Akt signaling activity in HDFs.

**Conclusion:**

Fisetin promotes skin wound healing and attenuates proliferative scar formation by reducing inflammation, improving collagen remodeling, and suppressing fibrotic signaling. Mechanistically, fisetin upregulates PTEN and inhibits the PI3K/Akt/TGF-β1 axis, thereby limiting fibroblast activation and pathological extracellular matrix deposition. These findings suggest fisetin as a promising therapeutic candidate for wound management and scar prevention.

## 1 Introduction

Skin wound healing is a highly coordinated and dynamic biological process involving inflammation, cell proliferation, extracellular matrix (ECM) deposition and tissue remodeling (Liu et al., 2025; Singer, 2022). Although most acute wounds can heal smoothly, the disorder of the repair process often leads to over-activation of fibroblasts, abnormal deposition of collagen and the formation of proliferative scars (Fang et al., 2025). This kind of pathological scar will not only damage skin function and beauty, but also bring a heavy physical and mental burden to patients (Szlachcikowska et al., 2025). At the cellular level, fibroblasts play a key role in the synthesis and remodeling of ECM. In the process of physiological healing, type III collagen (COL3A) is first deposited to provide a flexible scaffold, and then gradually transitions to type I collagen (COL1A) to restore the tensile strength of the tissue (Mathew-Steiner et al., 2021). However, persistent myofibroblast activation, characterized by sustained α-smooth muscle actin (α-SMA) expression and excessive COL1A deposition, is a hallmark of fibrotic wound healing and hypertrophic scar formation (Kohlhauser et al., 2024). Therefore, the treatment strategy aimed at rebalancing collagen remodeling and limiting fibrosis remains a major challenge in wound management.

The phosphatidylinositol 3-kinase (PI3K)/Akt signaling pathway is a central regulator of cell survival, proliferation, migration and ECM remodeling. Its abnormal activation is closely related to the activation of fibroblasts and pathological fibrosis in various tissues (Huang et al., 2024; Li et al., 2023; Liu et al., 2024). Notably, PI3K/Akt signaling interacts with transforming growth factor-beta1 (TGF-β1), a master profibrotic cytokine that drives myofibroblast differentiation and collagen overproduction, thereby forming a central regulatory axis in fibrotic wound healing (Xiaojie et al., 2022). Therefore, regulating the PI3K/Akt signaling pathway has become a promising way to improve wound healing and inhibit excessive scar formation (Yin et al., 2022).

In recent years, natural products have attracted more attention as a source of therapeutic drugs with favorable safety and multi-target biological activities. Fisetin is a naturally occurring flavonoid, which is widely found in fruits and vegetables, and has a variety of pharmacological activities, including antioxidant, anti-inflammatory and anti-fibrotic effects (Grynkiewicz and Demchuk, 2019; Wu et al., 2022). Previous studies have shown that fisetin can regulate oxidative stress and fibrosis signaling pathways in a variety of disease models (Ju et al., 2023; Telrandhe et al., 2025). However, the therapeutic effects of fisetin on skin wound healing and the inhibition of proliferative scar formation remain unclear. Meanwhile, there is evidence that the PI3K/Akt/TGF-β1 axis plays a crucial role in regulating fibroblast activation and extracellular matrix remodeling (Fang et al., 2025). Therefore, we speculated that fisetin could promote wound healing and inhibit scar formation by modulating the PI3K/Akt/TGF-β1 axis. Network pharmacology provides a systematic framework for analyzing complex interactions between bioactive compounds, molecular targets and disease-related pathways (Liu et al., 2013). Combining it with molecular docking and experimental verification can deeply reveal the multi-target mechanism of natural products (Akter et al., 2025; Li et al., 2023; Zhang et al., 2024).

To elucidate the therapeutic role of fisetin in skin wound healing and scar regulation, we constructed *in vivo* and *in vitro* experimental models. The *in vivo* model utilized a full-thickness excisional wound model in rats, whereas the *in vitro* model employed human dermal fibroblasts (HDFs). This study aimed to systematically evaluate the effects of fisetin on fibroblast activation, collagen remodeling, and PI3K/Akt signaling during the wound repair process. In addition, the molecular mechanisms underlying fisetin-mediated regulation of extracellular matrix homeostasis and fibrotic responses were explored. The findings may provide mechanistic evidence supporting the potential application of fisetin as a novel therapeutic agent for promoting wound healing while limiting pathological scar formation.

## 2 Materials and methods

### 2.1 Reagents and antibodies

Fisetin and H_2_O_2_ were purchased from Shanghai Macklin Biochemical Technology Co., Ltd. (Shanghai, China). Dimethyl sulfoxide (DMSO), the Cell Counting Kit-8 (CCK-8), and the Calcein-AM/Propidium Iodide live/dead staining kit were purchased from Shanghai Beyotime Biotechnology Co., Ltd. (Shanghai, China). High-glucose Dulbecco’s modified Eagle’s medium/nutrient mixture F-12 (DMEM/F12), penicillin-streptomycin, and phosphate buffered saline (PBS) were purchased from Gibco (Thermo Fisher Scientific, USA). Fetal bovine serum was purchased from Bovogen (Victoria, Australia). SC79 and MK-2206 were purchased from TargetMol Chemicals Inc. (Shanghai, China). The antibodies against AKT, p-AKT and TGF-β1 were obtained from Epizyme Biotechnology Co., Ltd. (Shanghai, China). The antibodies against COL1A, COL3A, α-SMA and Goat Anti-Rabbit IgG (H + L) were obtained from Abmart Biotechnology Co., Ltd. (Shanghai, China). The glyceraldehyde-3-phosphate dehydrogenase (GAPDH) antibody was purchased from Servicebio Biotechnology Co., Ltd. (Wuhan, China).

### 2.2 Network pharmacology analysis

To explore the potential mechanism of fisetin in promoting wound healing and inhibiting scar formation, a systematic network pharmacology approach was employed. The chemical structure and SMILES identifier of fisetin were obtained from the PubChem database, and potential fisetin-related targets were predicted using the SwissTargetPrediction platform. Subsequently, targets related to wound healing and hypertrophic scar (HS) were collected by searching the GeneCards, Comparative Toxicogenomics Database, and Online Mendelian Inheritance in Man databases. All targets were integrated and deduplicated. The putative targets of fisetin were intersected with wound healing-related and HS-related targets, and the overlapping targets were identified using Venn diagram analysis performed with the Venny 2.1 online tool. To evaluate the importance of these common targets, the intersected targets were imported into the STRING 12.0 database (https://string-db.org/) for protein-protein interaction (PPI) network construction and topological analysis, with a combined score >0.7 as the screening criterion. To evaluate the network characteristics of each node, we calculated three topological parameters: degree, betweenness centrality, and closeness centrality. “Degree” indicates the number of direct interactions between a node and other nodes; “Betweenness Centrality” reflects the frequency with which the node acts as a bridge on the shortest path between other nodes; “Closeness Centrality” measures the average shortest distance from a node to all other nodes, indicating its information transmission efficiency. The higher these parameter values, the more important the node is in the network. We selected common targets whose topological parameter values were higher than the median as the key targets. Cytoscape software (version 3.10.3) was used to visualize and analyze the protein interaction network of fisetin on wound healing and scar formation to further clarify the core mechanism of the therapeutic effect of fisetin.

### 2.3 Functional enrichment analysis

Metascape is a comprehensive and authoritative bioinformatics platform that integrates multiple databases (http://metascape.org/) including Gene Ontology (GO) and Kyoto Encyclopedia of Genes and Genomes (KEGG). To further clarify the biological functions and signaling pathways of fisetin in wound healing and scar formation, we uploaded the key targets identified from the PPI network to the Metascape platform for enrichment analysis. We took “*Homo sapiens*” (human) as the parameter and carried out GO function annotation (including biological processes (BP), cell components (CC) and molecular function (MF)) and KEGG pathway enrichment analysis. Based on the correlation with physiological regulation and pharmacological mechanism, we chose the top-ranked GO terms and KEGG pathways. Finally, we used the WeiShengXin online platform (https://www.bioinformatics.com.cn/) to visualize the enrichment analysis results, which intuitively showed the relevant biological processes and signaling pathways.

### 2.4 Molecular docking validation

Based on the results of network pharmacology and topological analysis, AKT1 was identified as a core target involved in the PI3K/AKT signaling pathway and was selected for molecular docking validation. The three-dimensional crystal structure of AKT1 was retrieved from the RCSB Protein Data Bank (https://www.rcsb.org/). The two-dimensional chemical structure of fisetin was obtained from the PubChem database (https://pubchem.ncbi.nlm.nih.gov/) and subsequently converted into a three-dimensional structure using Chem3D software. Molecular docking was performed using AutoDock Vina to evaluate the binding affinity between fisetin and AKT1. A binding energy of ≤ −5.0 kcal/mol was considered indicative of favorable binding, with lower energy values representing stronger ligand-protein interactions. The docking conformations were visualized using PyMOL, and two-dimensional interaction diagrams were generated using Discovery Studio (2016 Client) to identify key amino acid residues involved in the interaction.

### 2.5 Cell culture and cell proliferation assay

HDFs were purchased from Shanghai QuiCell Biotechnology Co., Ltd. Cells were cultured in DMEM/F-12 medium supplemented with 10% fetal bovine serum and 1% penicillin/streptomycin at 37 °C in a humidified atmosphere containing 5% CO_2_. Cell proliferation was evaluated using CCK-8 according to the manufacturer’s instructions. HDFs were seeded in 96-well plates at a density of 5 × 10^3^ cells per well and allowed to adhere overnight. Cells were then treated with fisetin at concentrations of 5, 10, and 20 μM, while control cells received vehicle (≤0.1% DMSO). Cell viability was assessed at 1, 3, and 7 days after treatment. At each time point, the culture medium was replaced with 100 μL of fresh medium containing 10 μL of CCK-8 solution and incubated at 37 °C for 1–2 h in the dark. The absorbance was measured at 450 nm using a microplate reader (Infinite 200 Pro, Tecan, Switzerland). Relative cell viability was expressed as a percentage of the control group. All experiments were performed in triplicate.

### 2.6 Live/dead cell staining assay

To evaluate the cytotoxic effects of fisetin on HDFs, a Calcein-AM/Propidium Iodide live/dead staining kit was used according to the manufacturer’s instructions. HDFs were seeded in 24-well plates at a density of 1 × 10^4^ cells per well and treated with fisetin at concentrations of 5, 10, and 20 μM for 72 h (day 3). Control cells received vehicle (≤0.1% DMSO). After treatment, cells were washed with PBS and incubated with a staining working solution containing 2 μM Calcein-AM and 4 μM PI at 37 °C in the dark for 30 min. Fluorescence images were captured using a confocal laser scanning microscope (STELLARIS 5, Leica Microsystems, Germany). Viable cells exhibited green fluorescence under 488 nm excitation, while non-viable cells showed red fluorescence under 545 nm excitation, as specified by the staining kit.

### 2.7 Cell treatment and experimental grouping

HDFs were seeded in six-well plates and cultured until approximately 85%–90% confluence. Cells were randomly divided into eight groups and subjected to two treatment conditions. Under non-oxidative conditions, cells were treated with fisetin (5, 10, or 20 μM), which was dissolved in DMSO and diluted with serum-containing medium, with the final DMSO concentration maintained at ≤ 0.1%; control cells received vehicle alone, and all groups were incubated for 48 h. For oxidative stress experiments, the serum-containing medium was removed, cells were washed three times with sterile PBS and were incubated in serum-free medium for 24 h. Cells were then pretreated with the AKT activator SC79 or the AKT inhibitor MK-2206 (5 μM) for 1 h, followed by exposure to hydrogen peroxide (H_2_O_2_, 500 μM) for 1 h to induce oxidative stress, as previously described (Pan et al., 2019). After H_2_O_2_ exposure, cells were washed three times with PBS. Cells in the fisetin-treated groups were then incubated with 20 μM fisetin in serum-containing medium for an additional 24 h, whereas cells in the SC79 and MK-2206 groups received serum-containing medium with vehicle alone, prior to subsequent analyses.

### 2.8 Animal experiments

Adult male Sprague-Dawley rats (6–8 weeks old) were purchased from SPF (Beijing) Biotechnology Co., Ltd. (Beijing, China) and housed under standard laboratory conditions (23 °C ± 2 °C, 12 h light/dark cycle) with free access to food and water. After acclimatization, the rats were randomly divided into four groups (n = 6 per group): a vehicle control group and three fisetin-treated groups receiving 5, 10, or 20 μM fisetin, respectively. Rats were individually identified using a combination of non-invasive tail marking and dorsal fur shaving to ensure reliable identification while minimizing stress. Anesthesia was induced by intraperitoneal injection of 0.3 mL/100 g of 0.5% pentobarbital sodium. Following dorsal hair removal and povidone-iodine disinfection, a 2.0 cm diameter full-thickness excisional wound was created on the dorsal midline of each rat. Fisetin or vehicle solution (sterile saline containing ≤1% DMSO) was administered via subcutaneous injection (100 μL per rat) at sites adjacent to the wound margins, avoiding direct injection into the wound bed. Injections were performed once daily for seven consecutive days, with injection sites alternated to ensure uniform diffusion around the wound area. During the experiment, the wound healing was evaluated by macroscopic observation. Digital photographs were taken of the wound on day 0, 7, 14, and 21 after the operation to record the healing process. The wound area was measured using ImageJ software (NIH, Bethesda, MD, USA), and the wound healing rate was calculated according to the method described in a previous study (Chen et al., 2026) by the following formula: (wound area on day 0 − wound area on day *X*)/wound area on day 0% × 100%.

### 2.9 Immunohistochemical staining

Rats were euthanized on day 21, and wound tissues were harvested for subsequent histological and immunohistochemical analyses. Paraffin-embedded wound tissue sections were prepared for immunohistochemical analysis. Briefly, sections were deparaffinized in xylene and rehydrated through a graded ethanol series. Endogenous peroxidase activity was blocked by incubation with 0.3% hydrogen peroxide at room temperature for 20 min. Antigen retrieval was subsequently performed using citrate buffer under heat-induced conditions. After cooling, the sections were blocked with 5% bovine serum albumin for 30 min to reduce nonspecific binding. The sections were then incubated with primary antibodies against COL1A and COL3A at 4 °C overnight. After washing, the sections were incubated with the corresponding horseradish peroxidase (HRP)-labeled secondary antibodies at room temperature for 30 min 3,3′-diaminobenzidine was used as the chromogenic substrate for color development, and the sections were counterstained with hematoxylin. Finally, the sections were dehydrated, cleared, mounted, and observed under a light microscope. ImageJ software was used for quantitative analysis to measure the wound area and evaluate the expression levels of COL1A and COL3A by calculating the mean optical density (MOD) of immunohistochemically stained sections.

### 2.10 Hematoxylin and eosin (H&E) and Masson’s trichrome staining

On the 21st day, the wound tissue was collected, fixed with 4% paraformaldehyde, dehydrated by gradient ethanol series, paraffin-embedded, and sliced to a thickness of 4–5 μm. Hematoxylin and eosin (H&E) staining was carried out to evaluate the overall histopathological changes in the wound area. Commercial staining kits were used to carry out Masson three-color staining according to the manufacturer’s instructions to evaluate the collagen deposition. In Masson-dyed slices, collagen fibers were observed as blue-stained areas. Quantitative analysis of collagen deposition was performed using ImageJ software, and the collagen volume fraction (CVF) was calculated as the ratio of the collagen-positive area to the total tissue area within the wound region. Histological inflammation was semi-quantitatively evaluated on H&E-stained sections using a 0–4 scoring system (0, no inflammatory cell infiltration; 1, minimal infiltration; 2, mild inflammation; 3, moderate inflammation; 4, severe inflammation) (Kim et al., 2011). For each rat, five randomly selected high-power fields within the wound region were assessed at ×200 magnification. All sections were evaluated in a blinded manner by an experienced pathologist. The average score of five fields was calculated to obtain one value per animal (n = 6 per group) for subsequent statistical analysis.

### 2.11 Western blotting

After the indicated treatments, HDFs were lysed using RIPA buffer containing protease and phosphatase inhibitors (Beyotime Biotechnology, Shanghai, China) and incubated on ice for 30 min. The cell lysates were then centrifuged at 12,000 rpm for 10 min at 4 °C, and the protein concentration of the supernatant was determined using a BCA protein assay kit (Beyotime Biotechnology, Shanghai, China).

Equal amounts of protein were separated by 10% sodium dodecyl sulfate-polyacrylamide gel electrophoresis (SDS-PAGE) and transferred onto polyvinylidene fluoride (PVDF) membranes (Millipore, Billerica, MA, USA). The membranes were blocked with TBST containing 5% non-fat milk for 1 h at room temperature and then incubated overnight at 4 °C with primary antibodies against COL1A (1:1000), COL3A (1:1000), TGF-β1 (1:2000), α-SMA (1:2000), AKT (1:1000), p-AKT (1:1000), and GAPDH (1:1000). After washing three times with TBST, the membranes were incubated with HRP-conjugated secondary antibodies (1:5000) for 1 h at room temperature. Protein bands were visualized using an enhanced chemiluminescence detection kit (Epizyme, Shanghai, China). Band intensities were quantified using ImageJ software and normalized to GAPDH. All experiments were independently repeated three times.

### 2.12 RT-qPCR

Reverse transcription-quantitative polymerase chain reaction (RT-qPCR) analysis was performed as previously reported (Qin et al., 2026). Total RNA was extracted from treated HDFs and rat wound tissues using TRIzol reagent (Beyotime, China), and cDNA synthesis was performed using SuperMix (Yeason, China). RT-qPCR was conducted using SYBR Green Master Mix (Yeason, China) on a real-time PCR system. Species-specific primers were designed for human (*H. sapiens*) and rat (*Rattus norvegicus*) genes, respectively. Human-specific primers were used for HDF experiments, whereas rat-specific primers were applied to wound tissue samples. The expression levels of fibrosis-related genes were determined in a species-dependent manner. In HDFs, COL1A1, COL3A1, TGFB1, and ACTA2 were analyzed, whereas Pten, Tgfb1, and Acta2 were evaluated in rat wound tissues. GAPDH/Gapdh served as the internal controls. Each gene was measured in triplicate and the relative change in gene expression was calculated with the 2^−ΔΔCT^ method. The sequences of primers used in this study are listed in Table 1.

**TABLE 1:** Species-specific primer sequences used for RT-qPCR.

Gene	Species	Forward (5′–3′)	Reverse (5′–3′)
GAPDH	Human	CTG​CCA​ACG​TGT​CAG​TGG​TG	TCA​GTG​TAG​CCC​AGG​ATG​CC
TGFB1	Human	TTG​CTT​CAG​CTC​CAC​AGA​GA	TGG​TTG​TAG​AGG​GCA​AGG​AC
ACTA2	Human	GTT​CCG​CTC​CTC​TCT​CCA​AC	GTG​CGG​ACA​GGA​ATT​GAA​GC
COL1A1	Human	CCT​GGA​TGC​CAT​CAA​AGT​CT	AAT​CCA​TCG​GTC​ATG​CTC​TC
COL3A1	Human	GGA​GCT​GGC​TAC​TTC​TCG​C	GGG​AAC​ATC​CTC​CTT​CAA​CAG
Gapdh	Rat	CGC​TAA​CAT​CAA​ATG​GGG​TG	TTG​CTG​ACA​ATC​TTG​AGG​GAG
Pten	Rat	ACC​AAT​GGC​TAA​GTG​AAG​ACG​A	CAG​GGC​CTC​TTG​TGC​CTT​TA
Tgfb1	Rat	TGA​GTG​GCT​GTC​TTT​TGA​CG	TGG​GAC​TGA​TCC​CAT​TGA​TT
Acta2	Rat	GTC​CCA​GAC​ATC​AGG​GAG​TAA	TCG​GAT​ACT​TCA​GCG​TCA​GGA

### 2.13 Statistical analysis

All statistical analyses were performed using SPSS 27.0. Data normality was assessed using the Shapiro-Wilk test, and homogeneity of variances was evaluated by Levene’s test. Continuous variables were expressed as the mean ± standard deviation. Differences among multiple groups were analyzed using one-way analysis of variance (ANOVA). When appropriate, two-way ANOVA was applied to evaluate the effects of two independent factors and their interaction. If a statistically significant difference was detected, Tukey’s honestly significant difference post hoc test was used for pairwise comparisons. For non-normally distributed data, the Kruskal–Wallis test followed by Dunn’s multiple comparisons test was employed. Graphical representations were generated using GraphPad Prism version 10.1.2. A *P*-value of less than 0.05 was considered statistically significant.

## 3 Results

### 3.1 Identification of potential targets of fisetin in wound healing and scar formation

**FIGURE 1:**
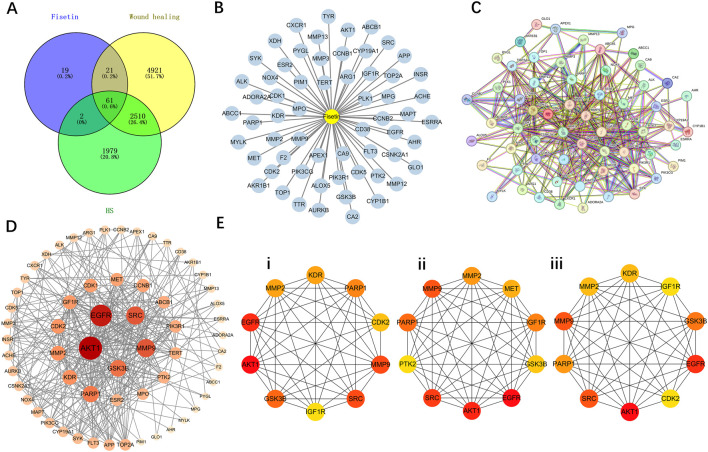
Common targets of fisetin in wound healing and hypertrophic scar (HS) and their protein-protein interaction (PPI) network analysis. **(A)** Venn diagram showing the common targets of fisetin, wound healing and HS. **(B)** Visualization of the network of common targets of fisetin, wound healing and HS. **(C)** Protein-protein interaction network of the common targets of fisetin, wound healing and HS. **(D)** Topological analysis network of target protein interaction. **(E)** CytoHubba analysis of potential target PPI network: **(I)** Degree; **(ii)** Maximal Clique Centrality (MCC); **(iii)** Maximum Neighborhood Component (MNC).

The predicted targets of fisetin were intersected with disease-related targets associated with wound healing and HS. As shown in Figure 1A, Venn diagram analysis revealed a subset of overlapping targets shared by fisetin, wound healing and HS. These common targets were considered potential therapeutic targets of fisetin and were selected for subsequent protein-protein interaction and network analyses.

### 3.2 PPI network construction and key target screening

The common targets were visualized as a drug-target interaction network using Cytoscape, in which fisetin was positioned at the center and the 61 overlapping targets associated with wound healing and hypertrophic scar were distributed around it, as shown in Figure 1B. This network illustrates the potential interactions between fisetin and its predicted therapeutic targets.

To further analyze the interactions, we built a well-defined PPI network with common targets (Figure 1C). Then, the topology analysis of the PPI network was carried out to evaluate the importance of each node in the network. As shown in Figure 1D, multiple targets showed high topological parameter values, indicating that they play a central role in the network. To further screen the most critical central targets, we used three algorithms for CytoHubba analysis: Degree, Maximal Clique Centrality (MCC) and Maximum Neighborhood Component (MNC). The top-ranked targets identified by each algorithm are shown in Figure 1E (i-iii). Several key proteins, including serine/threonine kinase 1 (AKT1), epidermal growth factor receptor (EGFR), SRC proto-oncogene, non-receptor tyrosine kinase (SRC), matrix metalloproteinase-9 (MMP-9), matrix metalloproteinase-2 (MMP-2), glycogen synthase kinase 3 beta (GSK3β), Poly (ADP-ribose) polymerase 1 (PARP1), cyclin-dependent kinase 2 (CDK2) and kinase insert domain receptor (KDR), were consistently identified as hub genes in different algorithms. This suggests that these targets may play a key role in the regulatory effects of fisetin on wound healing and hypertrophic scar formation.

### 3.3 GO and KEGG enrichment analysis of key targets

**FIGURE 2:**
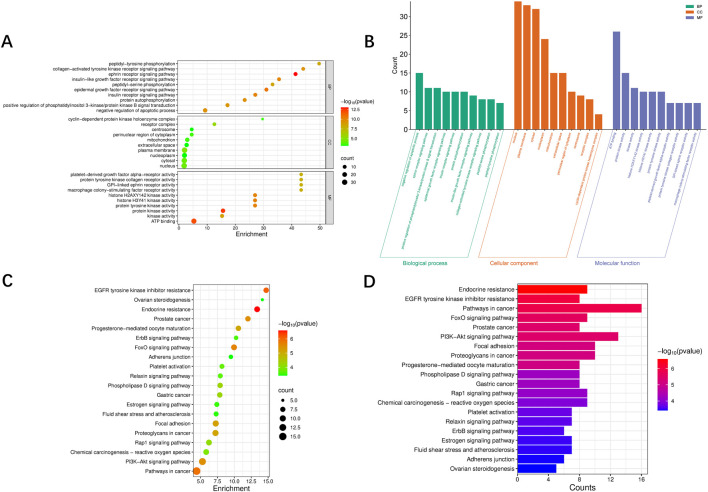
Enrichment analysis. **(A,B)** Gene Ontology (GO) enrichment of common targets of fisetin, wound healing, and HS. **(C,D)** Kyoto Encyclopedia of Genes and Genomes (KEGG) enrichment of common targets of fisetin, wound healing, and HS.

To further clarify the biological function and signaling pathways of the key targets of fisetin, we conducted GO and KEGG enrichment analysis. GO enrichment analysis shows that these key targets are mainly involved in biological processes related to protein phosphorylation, receptor tyrosine kinase signaling and oxidative stress response. In terms of cellular components, these targets are mainly enriched in the plasma membrane, cytoplasm, nucleus and extracellular space; and molecular function analysis shows that these targets are significantly enriched in protein kinase activity, ATP binding and receptor signal conduction activity, as shown in Figures 2A–C.

The enrichment analysis of KEGG pathways further reveals that these key targets are significantly enriched in pathways related to cell adhesion and migration, including focal adhesion and adherens junction, as well as pathways associated with angiogenesis and inflammatory regulation, such as Rap1 signaling pathway and platelet activation pathway. It is worth noting that the PI3K/Akt signaling pathway also appears in the enrichment pathway, indicating that it may be involved in the regulatory effect of fisetin on tissue repair and wound healing (Figure 2D). In addition, pathways related to oxidative stress regulation and cell survival, including the FoxO signaling pathway and the chemical carcinogenesis–reactive oxygen species, are also enriched. In summary, these results suggest that fisetin may play a therapeutic role by coordinating multiple signaling pathways involved in tissue repair and wound healing.

### 3.4 Molecular docking analysis of fisetin with AKT1

Based on the results of network pharmacology and enrichment analysis, AKT1 was identified as a core target closely associated with the PI3K/AKT signaling pathway and wound healing-related biological processes. To further evaluate the potential interaction between fisetin and AKT1, molecular docking analysis was performed. Docking simulation was performed using the crystal structure of AKT1 (Protein Data Bank ID: 3O96). The docking results demonstrated that fisetin exhibited a favorable binding affinity toward AKT1, with a predicted binding energy of −10.35 kcal/mol, indicating a stable ligand-protein interaction.

**FIGURE 3:**
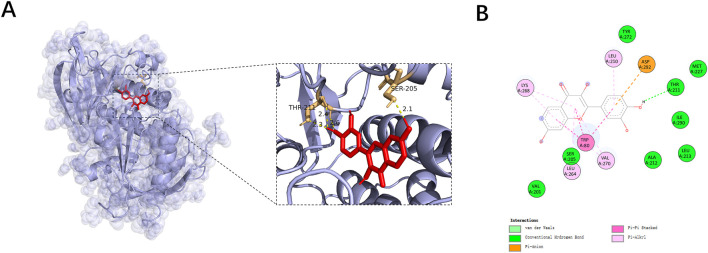
Molecular docking analysis of fisetin with serine/threonine kinase 1 (AKT1). **(A)** Three-dimensional binding conformation of fisetin within the active pocket of AKT1. **(B)** Two-dimensional interaction diagram illustrating hydrogen bonds and hydrophobic interactions between fisetin and surrounding amino acid residues.

As shown in Figure 3, fisetin was well accommodated within the active pocket of AKT1. Detailed interaction analysis revealed that fisetin formed conventional hydrogen bonds with key residues such as Thr211 and Ser205, along with multiple hydrophobic interactions involving Val201, Leu210, Ile290, and other surrounding amino acids. These interactions contribute to the stabilization of the fisetin–AKT1 complex. Collectively, these findings provide structural evidence supporting the direct interaction between fisetin and AKT1, thereby reinforcing the hypothesis that fisetin may exert its biological effects through modulation of the PI3K/AKT signaling pathway.

### 3.5 Effects of fisetin on HDF proliferation and cytotoxicity

**FIGURE 4:**
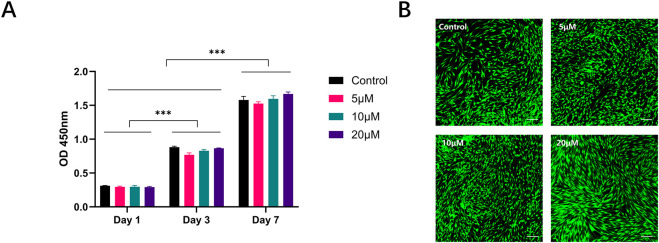
Effects of fisetin on HDF proliferation and cytotoxicity. **(A)** Cell proliferation of HDFs treated with fisetin (5, 10, and 20 μM) for 1, 3, and 7 days, as evaluated by CCK-8 assay. ***p < 0.001. **(B)** Live/dead staining of HDFs after 3 days of fisetin treatment. Scale bar = 100 μm.

To investigate the influence of fisetin on HDF proliferation, cell viability was assessed using the CCK-8 assay at 1, 3, and 7 days. As shown in Figure 4A, the OD values increased over time in all groups, reflecting the normal growth of HDFs during culture. Statistical analysis demonstrated a significant effect of incubation time on cell viability (*p* < 0.001). In contrast, treatment with fisetin at concentrations ranging from 5 to 20 μM did not produce obvious changes in cell viability compared with the control group.

Live/dead staining was further performed on day 3 to evaluate potential cytotoxic effects (Figure 4B). Most cells exhibited green fluorescence, with only a small number of red-stained cells observed across all groups. Cell morphology remained spindle-shaped and comparable to that of the control group. These findings indicate that fisetin at concentrations up to 20 μM did not exert detectable cytotoxic effects on HDFs under the present experimental conditions.

### 3.6 Fisetin promotes wound healing in a rat full-thickness excisional wound model

Based on the network pharmacology and molecular docking analyses, *in vivo* experiments were subsequently conducted to evaluate the therapeutic effects of fisetin on wound healing. Representative macroscopic images showed that from day 0 to day 21, the wounds in all groups gradually closed; however, compared with the control group, the wound healing speed of the fisetin treatment group was faster (Supplementary Figure S1A). Quantitative analysis showed that fisetin treatment significantly improved the wound healing rate on day 7, among which the 20 μM group showed a more significant effect than the control group (Supplementary Figure S1C). By the 14th day, the wound healing rate of the fisetin treatment group showed an accelerated trend, but no statistically significant differences were observed between the groups. By the 21st day, the wounds of all groups were almost completely closed. These results showed that fisetin treatment, especially at higher concentrations, can effectively promote early wound healing *in vivo*.

### 3.7 Fisetin improves histological repair, attenuates inflammation, and regulates collagen remodeling *in vivo*

To further evaluate the effect of fisetin on tissue repair, we conducted a histological evaluation. Masson’s trichrome staining showed that the collagen deposition in the wound of the fisetin treatment group increased and the distribution was more uniform (Supplementary Figure S1A). Quantitative analysis confirmed that the treatment of fisetin significantly increased the CVF, of which the collagen deposition in the 20 μM group was the highest among all groups (Supplementary Figure S1D). The results of H&E staining showed that compared with the control group, the epithelial regeneration of the fisetin treatment group improved on the 21st day, and the tissue structure was more orderly. Moreover, inflammatory cell infiltration was markedly reduced in fisetin-treated groups, particularly in the 10 and 20 μM groups. Quantitative analysis of histopathological inflammation scores confirmed that fisetin treatment significantly alleviated inflammatory responses compared with the control group (Supplementary Figure S1E).

Immunohistochemical staining further showed that the treatment of fisetin significantly changed the composition of collagen in the wound tissue. Compared with the control group, the expression of COL1A in the fisetin treatment group was reduced and the expression of COL3A was enhanced, especially in the 10 and 20 μM groups (Supplementary Figure S1B). Quantitative analysis showed that the COL1A/COL3A ratio was significantly reduced in fisetin-treated groups compared with the control group (Supplementary Figure S1F), indicating that the collagen composition had changed in a direction that was more conducive to regeneration. These results showed that fisetin can promote wound healing and regulate collagen remodeling, which may reduce scar formation.

### 3.8 Fisetin regulates PTEN, TGF-β1, and α-SMA mRNA expression in wound skin tissues

To clarify whether fisetin affects fibrosis-related gene expression *in vivo*, RT-qPCR was performed to detect the mRNA levels of PTEN, TGF-β1, and α-SMA in wound tissues on day 21. As shown in Supplementary Figure S2A, fisetin significantly increased PTEN mRNA levels in a dose-dependent manner. The 10 μM and 20 μM groups exhibited higher expression than the 5 μM group, with no difference between the two higher doses. Given that PTEN negatively regulates PI3K/AKT signaling, its upregulation may attenuate pro-fibrotic signaling.

In contrast, TGF-β1 mRNA levels were significantly reduced after fisetin treatment (Supplementary Figure S2B). All treatment groups exhibited lower expression than the control group, with stronger inhibition observed at 10 μM and 20 μM. Consistent with these findings, α-SMA mRNA expression was also significantly decreased in the fisetin-treated groups (Supplementary Figure S2C). The suppression became more evident with increasing concentrations, and the lowest level was detected in the 20 μM group. Taken together, fisetin increased PTEN expression while decreasing TGF-β1 and α-SMA transcription in wound tissues at day 21, indicating a potential inhibitory effect on fibrotic remodeling during the later stage of wound healing.

### 3.9 Fisetin attenuates profibrotic marker expression and promotes regenerative collagen remodeling in HDFs

**FIGURE 5:**
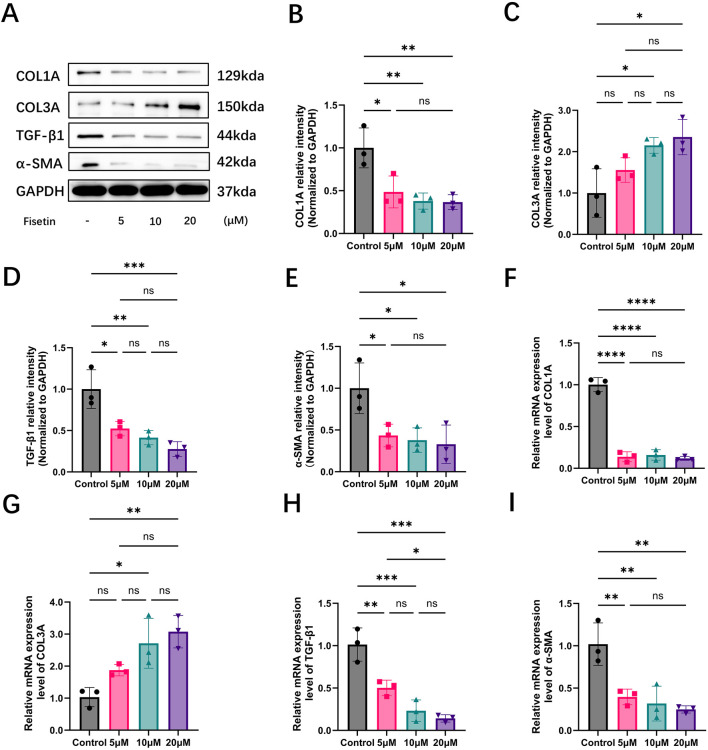
Fisetin regulates collagen-related marker expression in human dermal fibroblasts (HDFs) under basal conditions. **(A)** Representative Western blot images showing the protein expression of COL1A, COL3A, transforming growth factor-beta1 (TGF-β1), alpha-smooth muscle actin (α-SMA), and GAPDH in HDFs treated with fisetin (5, 10, and 20 μM) under basal (non-oxidative) conditions. **(B–E)** Quantitative analysis of COL1A, COL3A, TGF-β1, and α-SMA protein expression normalized to GAPDH. **(F–I)** Relative mRNA expression levels of COL1A1, COL3A1, TGF-β1, and α-SMA determined by RT-qPCR in HDFs following fisetin treatment. (*p < 0.05, **p < 0.01, ***p < 0.001, ****p < 0.0001; ns, not significant).

To investigate the dose-dependent effects of fisetin on collagen remodeling-related markers in HDFs under basal conditions, cells were treated with fisetin at concentrations of 5, 10, and 20 μM. Western blot analysis demonstrated that fisetin treatment markedly reduced the protein expression of profibrotic markers, including COL1A, TGF-β1, and α-SMA, while progressively increasing COL3A expression compared with the control group (Figure 5A). These results indicate that fisetin exerts a direct regulatory effect on fibroblast-associated fibrotic and regenerative markers under non-oxidative conditions.

Densitometric quantification showed that COL1A protein levels were significantly decreased following fisetin treatment, with a pronounced reduction observed at 5 μM and sustained suppression at higher concentrations (Figure 5B). In contrast, COL3A protein expression exhibited a concentration-dependent increase, reaching statistical significance at 10 and 20 μM compared with the control group (Figure 5C). In parallel, fisetin significantly downregulated TGF-β1 protein expression, with the lowest levels detected in the 20 μM treatment group (Figure 5D). Similarly, α-SMA protein expression was markedly reduced by fisetin, indicating suppression of myofibroblast-associated phenotypic markers (Figure 5E).

Consistent with the protein expression patterns, RT-qPCR analysis demonstrated that fisetin significantly decreased the mRNA expression of COL1A1, TGF-β1, and α-SMA, while markedly upregulating COL3A1 expression in a dose-dependent manner (Figures 5F–I). Notably, transcriptional repression of COL1A1 and TGF-β1 was evident even at lower fisetin concentrations, whereas maximal induction of COL3A1 occurred at 10 and 20 μM.

In conclusion, these results indicated that fisetin modulates collagen remodeling in HDFs under basal conditions by downregulating profibrotic markers, including COL1A, TGF-β1, and α-SMA, while simultaneously promoting the expression of regenerative collagen COL3A at both the protein and transcriptional levels.

### 3.10 Fisetin regulates AKT signaling and profibrotic marker expression under oxidative stress

**FIGURE 6:**
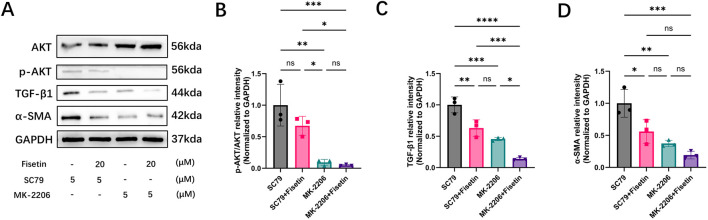
Involvement of the PI3K/Akt signaling pathway in fisetin-mediated regulation of fibrotic markers in HDFs. **(A)** Representative Western blot images showing the expression of AKT, p-AKT, TGF-β1, α-SMA, and GAPDH in HDFs treated with the AKT activator SC79 or the AKT inhibitor MK-2206 in the presence or absence of fisetin. **(B)** Quantitative analysis of p-AKT/AKT ratio normalized to GAPDH. **(C)** Quantitative analysis of TGF-β1 protein expression normalized to GAPDH. **(D)** Quantitative analysis of α-SMA protein expression normalized to GAPDH. (*p < 0.05, **p < 0.01, ***p < 0.001, ****p < 0.0001; ns, not significant).

To determine whether the antifibrotic effects of fisetin under oxidative stress are associated with AKT signaling, HDFs were exposed to H_2_O_2_ and treated with the AKT activator SC79 or the AKT inhibitor MK-2206, with or without fisetin. AKT, p-AKT, TGF-β1, and α-SMA protein levels were assessed by Western blotting (Figure 6A).

SC79 treatment significantly increased AKT phosphorylation, as reflected by an elevated p-AKT/AKT ratio, whereas MK-2206 markedly suppressed AKT activation (Figure 6B). Co-treatment with fisetin attenuated SC79-induced AKT phosphorylation but did not further reduce p-AKT levels in the presence of MK-2206.

Consistent with AKT activity, SC79 markedly increased the expression of TGF-β1 and α-SMA, while fisetin significantly reduced their upregulation (Figures 6C,D). MK-2206 treatment significantly decreased TGF-β1 and α-SMA expression, and fisetin did not exert additional inhibitory effects under AKT blockade. Collectively, these data indicated that fisetin influenced AKT activation and the expression of profibrotic markers such as TGF-β1 and α-SMA in HDFs exposed to oxidative stress.

## 4 Discussion

In the present study, a systematic experimental strategy was employed to investigate the therapeutic potential of fisetin in skin wound healing and scar regulation, integrating network pharmacology analysis with *in vivo* wound healing models and *in vitro* HDFs experiments. Attention was given to the regulatory effects of fisetin on fibroblast activation, extracellular matrix remodeling, and key profibrotic signaling pathways. These parameters are essential for evaluating both the efficacy and mechanistic basis of candidate agents intended for wound repair and scar prevention.

Network pharmacological analysis showed that fisetin exerts its effects through multiple targets closely related to wound repair and fibrosis, including AKT1, EGFR, SRC, MMP2 and MMP9. These targets are involved in key biological processes such as extracellular matrix remodeling, cell adhesion, oxidative stress regulation and intracellular signal transduction (Rajendran, 2024; Zhang et al., 2024). Consistent with these observations, GO and KEGG enrichment analysis identified several pathways related to tissue repair and fibrosis regulation, including focal adhesion, PI3K/Akt signaling pathway, Rap1 signaling pathway and oxidative stress-related pathway (Huang et al., 2023; Nakamura and Parkhurst, 2023; Wang et al., 2023). Considering the established crosstalk between PI3K/Akt signaling and TGF-β1, a key profibrotic mediator of fibroblast activation and collagen synthesis, our findings raise the possibility that fisetin may attenuate fibrosis through coordinated modulation of the PI3K/Akt/TGF-β1 signaling axis.

TGF-β1 is a key profibrotic cytokine that promotes myofibroblast differentiation and extracellular matrix accumulation, while PI3K/Akt signaling is critically involved in cell survival, proliferation, and fibrogenic responses. Accumulating evidence indicates that crosstalk between PI3K/Akt and TGF-β1 pathways plays a central role in fibroblast activation and pathological fibrosis (Tang et al., 2023; Yao et al., 2024). In the present study, fisetin upregulated PTEN expression, suppressed PI3K/Akt activation under oxidative stress conditions, and consistently reduced TGF-β1 expression in both *in vivo* and *in vitro* models, suggesting that its anti-fibrotic effects may be mediated through coordinated modulation of the PTEN/PI3K/Akt/TGF-β1 signaling axis.

Given the pivotal role of TGF-β1 in driving extracellular matrix production, inhibition of this signaling cascade would be expected to influence collagen remodeling. Excessive extracellular matrix deposition is also a hallmark feature of pathological scar formation. Dysregulated collagen synthesis, especially an increased COL I/COL III ratio, contributes to excessive fibrosis and impaired tissue remodeling (Limandjaja et al., 2021; Lin and Lai, 2024). In this study, fisetin treatment effectively attenuated fibroblast activation and modulated collagen remodeling, suggesting a favorable role in restoring extracellular matrix homeostasis during wound repair. These findings are consistent with previous reports describing the anti-fibrotic properties of flavonoids in skin-related pathological conditions (Liu et al., 2025).

Notably, TGF-β1 is derived not only from activated fibroblasts but also from inflammatory and immune cells within the wound microenvironment. Therefore, modulation of immune responses may constitute an upstream mechanism underlying the antifibrotic effects of fisetin. During the wound healing process, immune cells-particularly macrophages and mast cells-shape the local microenvironment by releasing a variety of cytokines and growth factors. Sustained M1 macrophage activation and excessive production of pro-inflammatory mediators such as TNF-α and IL-6 can prolong the inflammatory phase, which in turn enhances fibroblast activation and promotes fibrotic remodeling (Wynn and Vannella, 2016). Mast cells also participate in scar development through the release of histamine, tryptase, and TGF-β1, thereby facilitating myofibroblast differentiation and collagen accumulation (Gailit et al., 2001). Previous studies have reported that fisetin exhibits anti-inflammatory activity through modulation of signaling pathways such as PI3K/Akt and NF-κB, and by influencing macrophage polarization and cytokine secretion (Sun et al., 2021; Wu et al., 2022). In this study, fisetin significantly reduced histological inflammation scores and attenuated inflammatory cell infiltration in wound tissues. These findings support the notion that modulation of the inflammatory microenvironment contributes to the antifibrotic and wound-healing effects of fisetin.

Under oxidative stress conditions, which more closely mimic the pathological wound microenvironment, fisetin markedly attenuated AKT phosphorylation and reduced the expression of downstream profibrotic mediators, including TGF-β1 and α-SMA. Pharmacological modulation of AKT activity further supported the involvement of AKT signaling in fisetin-mediated antifibrotic effects. Activation of AKT by SC79 markedly increased p-AKT levels and upregulated the profibrotic markers TGF-β1 and α-SMA, whereas co-treatment with fisetin significantly attenuated these SC79-induced effects. In contrast, inhibition of AKT with MK-2206 suppressed p-AKT phosphorylation and reduced TGF-β1 and α-SMA expression, closely mimicking the effects of fisetin. Notably, additional fisetin treatment did not further enhance the inhibitory effects of MK-2206 (Figure 6). These findings indicate that fisetin suppresses fibroblast activation and fibrotic marker expression predominantly through modulation of AKT-dependent TGF-β1 signaling.

From the perspective of translational medicine, effective wound management requires not only rapid closure, but also the control of extracellular matrix remodeling to minimize fibrosis and pathological scar formation (Yang et al., 2025; Zhou et al., 2023). The results of this study show that fisetin may play a role in both aspects by regulating fibroblast activation, collagen composition and the PI3K/Akt/TGF-β1 signaling pathway in a specific environment-dependent manner. The multi-target regulatory properties of fisetin may be superior to single-pathway interventions, because the latter often cannot fully address the complexity of wound healing (Shen et al., 2025). Although further research is needed to optimize the dosing strategy, dosage scheme and long-term safety, this study provides mechanistic evidence to support the notion that fisetin, as an adjuvant treatment, may improve the quality of wound repair and reduce the risk of proliferative scar formation.

However, certain limitations remain in our research. First, the present study primarily focused on early and intermediate stages of wound healing, and long-term studies are required to further evaluate the sustained effects of fisetin on scar maturation and remodeling. Second, although the PI3K/Akt/TGF-β1 axis was identified as a key regulatory pathway, future studies are required to confirm this mechanism via gene knockout or antibody-blocking experiments.

## 5 Conclusion

This study provides comprehensive evidence that fisetin promotes skin wound healing and inhibits the formation of proliferative scars through multi-target and multi-pathway mechanisms. Fisetin improves the speed and quality of wound repair by regulating collagen remodeling and inhibiting fibroblast activation. The PI3K/Akt/TGF-β1 signaling pathway plays a key role in mediating these effects. These findings not only clarify the molecular basis of the therapeutic effect of fisetin, but also support its development as a safe and effective agent for wound management and scar prevention.

## Data Availability

The raw data supporting the conclusions of this article will be made available by the authors, without undue reservation.

## Glossary

AKT1: serine/threonine kinase 1
α-SMA: alpha-smooth muscle actin
BP: biological process
CC: cellular component
CCK-8: Cell Counting Kit-8
CDK2: cyclin-dependent kinase 2
COL1A: type I collagen
COL3A: type III collagen
CVF: collagen volume fraction
DMEM: Dulbecco’s modified Eagle’s medium
DMSO: dimethyl sulfoxide
ECM: extracellular matrix
EGFR: epidermal growth factor receptor
GAPDH: glyceraldehyde-3-phosphate dehydrogenase
GO: Gene Ontology
GSK3β: glycogen synthase kinase 3 beta
HDFs: human dermal fibroblasts
H&E: hematoxylin and eosin
HRP: horseradish peroxidase
HS: hypertrophic scar
KDR: kinase insert domain receptor
KEGG: Kyoto Encyclopedia of Genes and Genomes
MF: molecular function
MMP-2: matrix metalloproteinase-2
MMP-9: matrix metalloproteinase-9
PARP1: poly (ADP-ribose) polymerase 1
PBS: phosphate buffered saline
PI3K: phosphatidylinositol 3-kinase
PPI: protein-protein interaction
PVDF: polyvinylidene fluoride
RT-qPCR: reverse transcription-quantitative polymerase chain reaction
ROS: reactive oxygen species
SRC: SRC proto-oncogene non-receptor tyrosine kinase
TGF-β1: transforming growth factor-beta1
