# Supplementary material for: Fisetin promotes skin wound healing and inhibits pathological scar formation through modulation of the PI3K/Akt/TGF-β1 signaling axis
Source: Front Pharmacol. 2026 Mar 27;17:1793847. doi: 10.3389/fphar.2026.1793847 (PMC13066317; doi:10.3389/fphar.2026.1793847)
Supplement: Supplementary file 1 [file Image1.pdf]

Supplementary Material

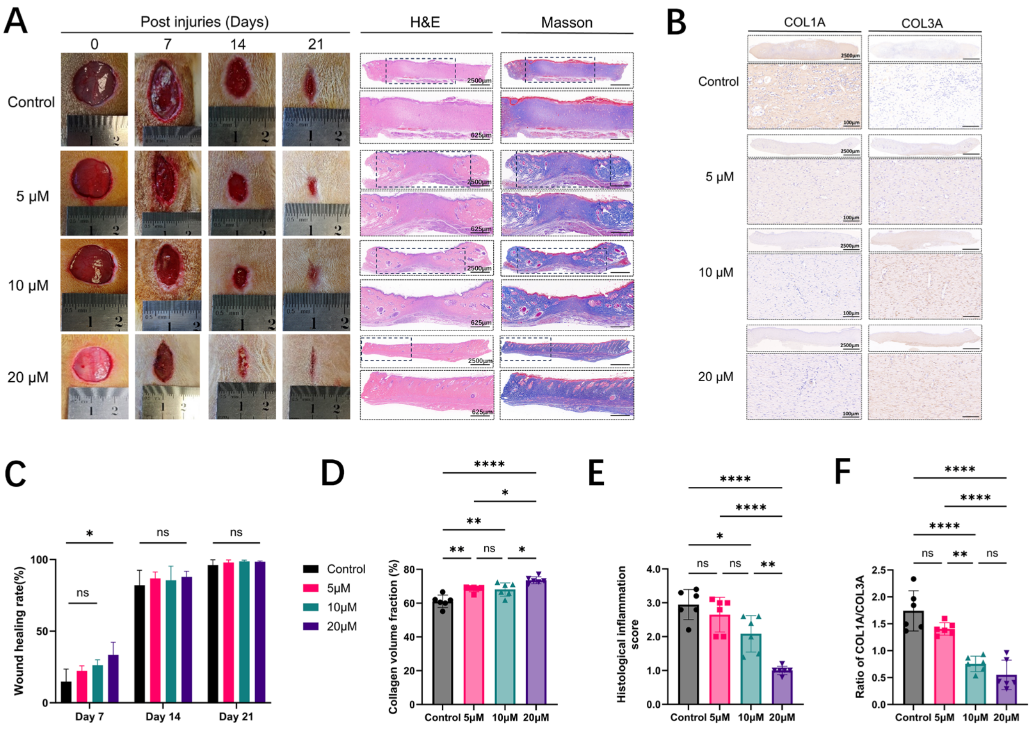

SUPPLEMENTARY FIGURE S1

Fisetin promotes wound healing and modulates collagen remodeling in a rat full-thickness excisional wound model. **(A)** Representative macroscopic images of wound healing in rats treated with vehicle (Control) or fisetin (5, 10, and 20 μM) on days 0, 7, 14, and 21 post-injury. Representative hematoxylin and eosin (H&E) staining and Masson's trichrome staining of wound tissues collected on day 21 are shown (n=6). Scale bars: 2500 μm (upper panels) and 625 μm (lower panels). **(B)** Representative immunohistochemical staining images of type I collagen (COL1A) and type III collagen (COL3A) in wound tissues on day 21. Scale bars: 2500 μm (upper panels) and 100 μm (lower panels) (n=6). **(C)** Quantitative analysis of wound healing rate on days 7, 14, and 21 (n=6). **(D)** Quantification of collagen volume fraction (CVF) based on Masson's trichrome staining (n=6). **(E)** Quantification of histological inflammation scores based on H&E staining (n=6). **(F)** Quantitative analysis of the COL1A/COL3A ratio determined from immunohistochemical staining (n=6). (**p* < 0.05, ***p* < 0.01, ****p* < 0.001, *****p* < 0.0001; ns, not significant.)

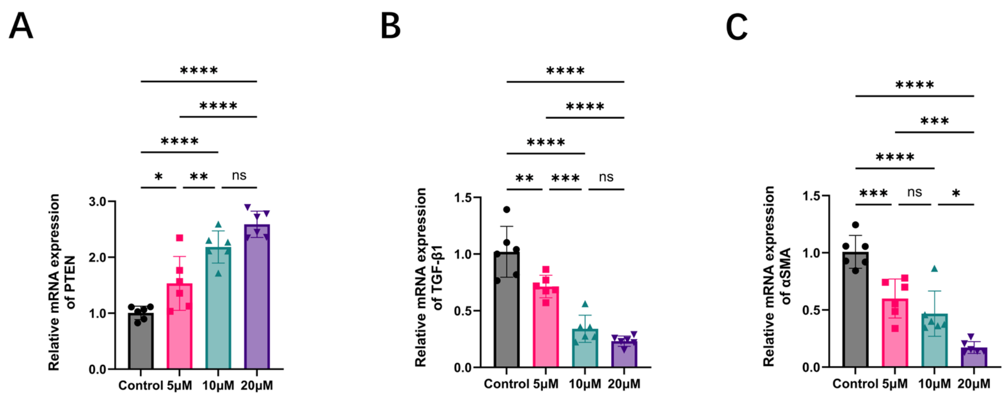

SUPPLEMENTARY FIGURE S2

Effects of fisetin on fibrosis-related gene expression in wound skin tissues. **(A-C)** Relative mRNA expression levels of PTEN, TGF-β1, and α-SMA in wound skin tissues on day 21 after fisetin treatment, as determined by RT-qPCR (n=6). (*$p < 0.05$, **$p < 0.01$, ***$p < 0.001$, ****$p < 0.0001$; ns, not significant.)
